# Couples coping in the community after the stroke of a spouse: A scoping review

**DOI:** 10.1002/nop2.413

**Published:** 2019-11-16

**Authors:** Sheena Ramazanu, Alice Yuen Loke, Vico Chung Lim Chiang

**Affiliations:** ^1^ The Hong Kong Polytechnic University Hung Hom Hong Kong; ^2^ Yishun Community Hospital Singapore Singapore; ^3^ School of Nursing The Hong Kong Polytechnic University Hung Hom Hong Kong

**Keywords:** community, coping, couples, experience, manage, nurses, nursing, stroke

## Abstract

**Aim:**

To summarize evidence on the poststroke coping experiences of stroke patients and spousal caregivers living at home in the community.

**Design:**

A scoping review.

**Methods:**

Extensive searches were conducted in credible databases. Articles published in the English language were retrieved. Data were extracted based on study location, aims, study design, sample size, time after stroke and key findings.

**Results:**

Out of 53 identified articles, 17 studies were included in the review. Five key themes were as follows: (a) emotional challenges; (b) role conflicts; (c) lack of strategies in coping; (d) decreased life satisfaction of the couples; and (e) marriage relationship: at a point of change. Couples were not sufficiently prepared to cope and manage with stroke at home on discharge from the hospital. This review emphasized the need for hospitals to implement policies to address the inadequate preparation of couples in coping with stroke.

## 1 INTRODUCTION

Stroke is a leading cause of morbidity and mortality globally. It is the fifth leading cause of death of people aged 15–59, claiming 6.2 million lives each year (World Health Organization, 2018). Stroke is a traumatic life event that affects not only the person who has a stroke but also their family caregivers (Singapore National Stroke Association, 2017). Stroke patients who live with disability will require assistance, creating challenges for the family members who care for them. In addition to physical, social and financial demands, the emotional impact of dealing with stroke can place tremendous stress on persons with stroke and their family caregivers (American Stroke Association, 2019).

The supportive care of a spousal caregiver has been considered pivotal to a stroke victim's recovery (Claesson, Gosman‐Hedstrom, Johannesson, Fagerberg, & Blomstrand, 2000; Persson, Ferraz‐Nunes, & Karlberg, 2012). Persons with stroke are usually looked after by their spouse, who gives them extensive support ranging from psychosocial to physical care (Adriaansen, Leeuwen, Visser‐ Meily, Bos, & Post, 2011). Six months after a stroke, approximately 4.6 hr per day is required to care for a person with stroke (Tooth, McKenna, Barnett, Prescott, & Murphy, 2005). With the addition of providing surveillance while caring, the hours required for caregiving could rise up to 14.2 hr per day (van Exel, Koopmanschap, van den Berg, Brouwer, & van den Bos, 2005). As stroke has an impact on both patients and their spouse, a better understanding of the experience of couples in coping with managing stroke at home is necessary. This would facilitate the provision of support when a stroke patient is discharged and the responsibility of caring for the patient is transferred from the hospital to the home. Traditionally, the term “spouse” refers to a husband or wife to whom one is legally married. The contemporary definition of a spouse now takes into account heterosexual common law relationships and partnerships (Ferlisi, 2001). The terms “spouse” and “partner” are used interchangeably in this review.

Current studies have examined the caregiver burden after a stroke (Rigby, Gubitz, & Phillips, 2009); the informational needs of stroke patients and their caregivers (Forster et al., 2012); the educational needs of stroke caregivers and persons with stroke (Hafsteinsdóttir, Vergunst, Lindeman, & Schuurmans, 2011); positive experiences of stroke caregiving (Mackenzie & Greenwood, 2012); and the rehabilitative experiences of stroke patients (Peoples, Satink, & Steultjens, 2011). However, there is a dearth of knowledge about coping with stroke at home in the community from the perspective of couples. The aim of this scoping review was to summarize the evidence on the poststroke coping experiences of spousal caregivers and persons with stroke living at home in the community. Such a review will provide the basis for recommending strategies for healthcare professionals and policymakers to consider when devising means to enhance essential discharge preparation procedures and support. The following research questions were raised for this review:

1. What is known from the existing literature about the coping experiences of couples in the community after the stroke of a spouse?
2. What is the impact on the spousal relationship after a stroke?

## 2 METHOD

The scoping review framework of Arskey and O'Malley (2005) was the method selected to underpin this review. Scoping reviews are conducted to understand the range and nature of research activity; to recognize gaps in the research literature; and to summarize key findings for practitioners who may lack the time or resources to carry out such work themselves (Arksey & O'Malley, 2005). The steps in conducting a scoping review include the following: (a) identifying the research questions; (b) identifying relevant studies, (c) selecting the studies; (d) charting the data; and (e) collating, summarizing and reporting the results (Arksey & O'Malley, 2005). A scoping review does not appraise the quality of the evidence but rather rigorously and transparently maps out key research areas (Arksey & O'Malley, 2005).

### 2.1 Identifying and selecting relevant studies

An extensive literature search was conducted from credible electronic databases. Primary databases that were used in the search for relevant articles included CINAHL, Embase, PubMed and Psych‐Info. The search covered studies published in the English language from 2008–2018. The primary focus of the review was on the experiences of couples coping with stroke in the community. The keywords used in the search were “stroke”, “couples”, “experience”, “coping”, “management” and “community”. The Boolean search was comprised of the following operators and keywords:

- Stroke OR cerebrovascular disease
- Spouse* OR partner OR (husband or wife)
- Coping OR experience* OR manage*
- Community OR home

### 2.2 Charting the data

**Figure 1:**
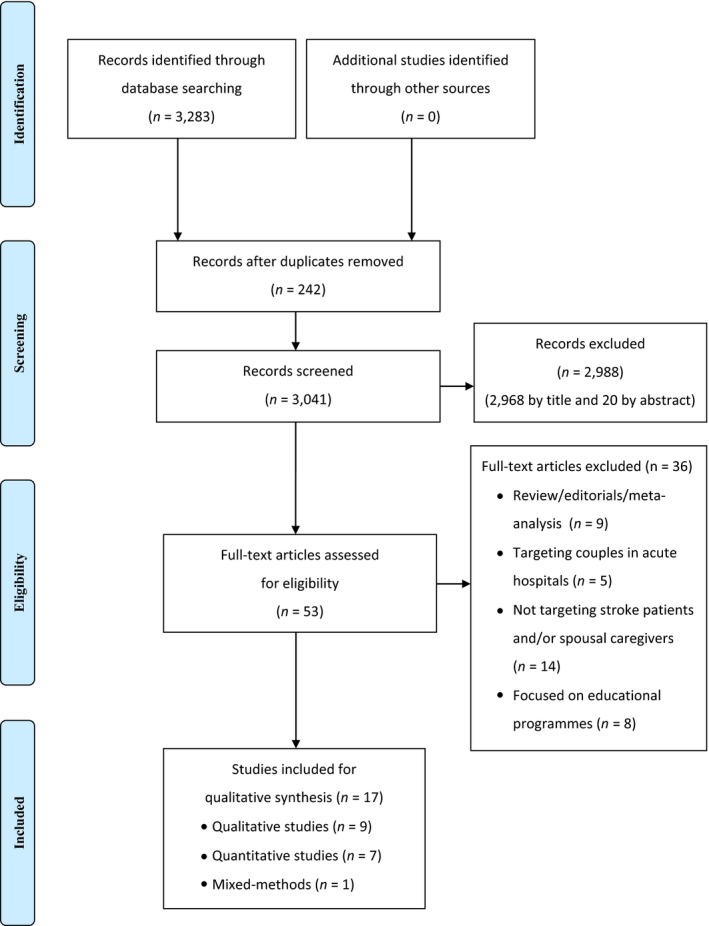
Literature screening and selection based on PRISMA

A total of 3,283 publications were identified via a search of the electronic databases. No additional records were identified through a hand or author search. Duplicates were removed with the aid of EndNoteX7 (*N* = 242). The remaining records (*N* = 3,041) were screened by title. A total of 2,968 articles were excluded, after which 73 articles remained. The abstracts of these 73 articles were screened, after which a further 20 articles were excluded. The full texts of the resulting 53 articles were then scrutinized using the criteria for inclusion in this scoping review. Thirty‐four articles were excluded for not meeting the inclusion criteria. In the end, a total of 17 articles, consisting of nine qualitative studies, seven quantitative studies and one mixed‐methods study, were included. The process of screening and selecting the articles for the review is shown in Figure 1.

### 2.3 Collating, summarizing and reporting results

An overview of the studies that were included in this review is given in Table 1. Data from the included studies were systematically extracted using the following headings: authors(s), year of publication, study location, aims of study, design, method, sample size, time after stroke and key findings. Drawing on the principles of thematic analysis put forward by Braun and Clarke (2006), the findings of the review were synthesized in relation to the scoping review questions. Familiarization with the studies was achieved first through reading and re‐reading. This facilitated the systematic organization of the findings from each study into meaningful clusters and their further categorization into the respective key findings in terms of themes.

**Table 1:** Overview of the studies

No	Author/date/location	Aims of study	Design/Methods	Sample size	Time after stroke	Key findings	Themes
Studies on couples coping poststroke at home							
1	Achten, Visser‐Meily, Post, and Schepers, (2012) Nieuwegein, The Netherlands	To compare the life satisfaction of stroke survivors and their spousal caregivers To examine spouses’ variables as determinants of the life satisfaction of patients	Cross‐sectional survey/questionnaires were administered	78 couples participated Mean age of stroke patients: 59 years Mean age of spousal caregivers: 55	3 years poststroke	More spouses (50%) as compared with patients (28%) were dissatisfied with life Couples experience decreased life satisfaction after stroke (chronic phase) There is a significant relationship between the life satisfaction of patients and that of stroke survivors	Decreased life satisfaction of the couples
2	Anderson et al. (2017) Alberta, Canada	To understand the key themes related to the reconstruction and breakdown of marriages poststroke	Qualitative/constructivist grounded theory/semi‐structured interviews were conducted	18 couples participated Mean age of stroke patients: 62.6 years Mean age of spousal caregivers: 62.3	Unspecified	Compatible role identities must be reconstructed to sustain marriages poststroke Factors that break down or aid in the reconstruction of marriages include, feeling overwhelmed, resolving conflicts, and perceiving value in the marriage	Emotional challenges Role conflicts Marriage relationship: at a point of change
3	Ekstam, Tham, & Borell (2011) Stockholm, Sweden	To identify and describe couples’ approaches to changes in everyday life 1 year poststroke	Qualitative/case study/interviews were conducted	2 couples Mean age of stroke patients: 78.5 years Mean age of spousal caregivers: 74.5 years	1 year poststroke	It was difficult for couples to engage in past activities Couples valued feedback to better understand their abilities and disabilities together Accepting the stroke situation and envisioning a future as a couple	Emotional challenges
4	Green and King (2009) Calgary, Canada	To explore male patients and their wife caregivers’ perceptions of factors that affect their quality of life and caregiver strain 1 year poststroke	Qualitative/semi‐structured interviews were conducted	26 couples Mean age of stroke patients: 63.9 years Mean age of spousal caregivers: 58.5	1 year postdischarge	Stroke couples felt vulnerable to the stroke situation; they felt that there was nothing they could do about it Feeling that they lacked information, lacked control, and were stigmatized Wife caregivers were hyper‐vigilant when monitoring the health and well‐being of stroke survivors	Emotional challenges Role conflicts
5	Godwin, Swank, Vaeth, and Ostwald (2013) Houston, USA	To examine the longitudinal dyadic relationship between caregivers’ and stroke survivors’ mutuality and caregivers’ and stroke survivors’ perceived stress	Longitudinal survey over 1 year/questionnaires were administered to the participants	159 stroke survivors and their spousal caregivers Mean age of stroke patients: 62.5 years Mean age of spousal caregivers: 60.6	12 months poststroke	The caregivers’ mutuality indicated an actor effect but not a partner effect The stroke survivors’ perceived stress showed a partner effect and affected the caregiver's perceived stress	Marriage relationship: at a point of change
6	McCarthy and Bauer (2015) Ohio, USA	To report findings from qualitative interviews of couples coping with stroke	Qualitative/structured survey followed by open‐ended individual interviews	31 couples (62 survivors and spouses) participated Mean age of stroke patients: 61.81 years Mean age of spousal caregivers: 60.42	1–36 months	‐ Four primary themes were identified: (1) practical and emotional challenges, (2) relationship challenges, (3) unexpected changes to the couples’ anticipated life course, and (4) mobilization of emotional and relationship resources after stroke	Emotional challenges
7	McCarthy and Lyons (2015) Ohio, USA	To investigate stroke survivors’ and caregiving spouses’ individual perspectives on survivor cognitive and physical functioning and the extent to which incongruence between the perceptions of the partners affects a spouse's depressive symptoms and overall mental health	Mixed‐methods/questionnaires were administered and interviews were conducted	35 couples participated Mean age of stroke patients: 60 years Mean age of spousal caregivers: 58 years	1–24 months poststroke	Quantitative data indicated that spouses rated survivor cognitive functioning as significantly worse than survivors rated their own, and that survivor spouse discrepancy scores for physical functioning were significantly associated with the spouse's depressive symptoms Qualitative data enhanced understanding about the nuances of partner incongruence and the ramifications of partner incongruence for the spouse's mental health	Emotional challenges
8	Ostwald et al. (2009) Houston, USA	To describe levels of stress in stroke survivors and spousal caregivers and identify predictors of stress in couples during their first year at home	Longitudinal survey over 1 year/questionnaires were administered	159 couples took part Mean age of stroke patients: 66.4 years Mean age of spousal caregivers: 62.5 years	3 months poststroke	Perceived Stress Scale (PSS) scores for stroke survivors and caregivers were positively correlated (*p*<.01) Preparation was the most powerful predictor of stress in caregivers	Emotional challenges
9	Quinn et al. (2014) Bolton, United Kingdom	To explore the experiences of couples when one partner has a stroke at a young age	Qualitative/semi‐structured interviews were conducted	8 couples took part Mean age of stroke patients: 51 years Mean age of spousal caregivers: 49.63 years	1–9 years poststroke	Stroke at a young age could disrupt couples’ lives There is a need for young couples to adapt and to reciprocate in their relationship roles through interventions	Emotional challenges Role conflicts
10	Rochette et al. (2007) Sherbrooke, Canada	To describe changes in the process of adaptation in the first 6 months after a stroke and to identify domains of the adaptation process in relation to participation and depressive symptoms for dyads	A short longitudinal survey over 6 months/questionnaires were administered to participants	88 individuals with first stroke and 47 spouses Mean age of stroke patients: 71.8 years Mean age of spousal caregivers: 61.2 years	2 weeks−6 months poststroke	Scores for the Threat, Challenge, and Stressfulness scales based on the Stress Appraisal Measure (SAM) decreased significantly for partners with stroke However, the score for the domain on the perceived uncontrollability of the situation increased significantly for spouses in the first 6 months	Emotional challenges Lack of strategies for coping
11	Visser‐Meily, Post, van de Port, van Heugten, & van den Bos, (2008) Maastricht, The Netherlands	To describe the psychosocial functioning of spouses at 1 and 3 years poststroke and to identify predictors of negative changes in psychosocial functioning	Longitudinal survey over 3 years/questionnaires were administered	119 stroke couples participated Mean age of stroke patients: 56 years Mean age of spousal caregivers: 53 years	1–3 years poststroke	51% reported a significant burden, 46% dissatisfaction with life, and 51% depressive symptoms Although the caregiver burden improved, life satisfaction, social support, and harmony in the relationship were significantly affected	Emotional challenges Decreased life satisfaction of the couples
12	Visser‐Meily et al., (2009) Maastricht, The Netherlands	To describe the psychosocial functioning of stoke spousal caregivers and examine their coping styles	Longitudinal survey over 3 years/questionnaires were administered	211 stroke couples participated Mean age of stroke patients: 56 years Mean age of spousal caregivers: 54 years	3 years poststroke	The depression score showed a non‐linear pattern with an initial decrease but a long‐ term increase amongst stroke spousal caregivers over 3 years poststroke	Emotional challenges Lack of strategies for coping
Studies on the experiences of spousal caregivers caring for stroke patients and coping at home							
13	Adriaansen, Leeuwen, Visser‐Meily, Bos, and Post (2011) Amsterdam, The Netherlands	To examine the course of social support for spousal caregivers and understand the associations between social support and life satisfaction	Prospective cohort study/questionnaires were administered	180 spouses participated Mean age of spousal caregivers: 53.3	2 months (T1), 1 year (T2), and 3 years (T3) poststroke discharge	Caregiver strain was associated with lower life satisfaction Social support was associated with higher life satisfaction	Decreased life satisfaction of the couples
14	Buschenfeld, Morris, and Lockwood (2009) Bristol, United Kingdom	To investigate the experiences of partners of young stroke survivors (<60 years old)	Qualitative/semi‐structured interviews were conducted	7 partners of stroke patients participated Mean age of spousal caregivers: 54.6	2–7 years poststroke	The stroke situation changed lives for stroke couples Couples endured effects on the self: trauma, isolation, growth They coped via emotion and problem‐focused coping Social support from friends and family is deemed to be crucial	Lack of strategies for coping
15	Gosman‐Hedstrom & Dahlin‐Ivanoff (2012) Goteborg, Sweden	To explore older women's experiences of their life situation and formal support as carers	Qualitative/focus group interviews were conducted	16 spousal caregivers participated Mean age of spousal caregivers: 74.3	2–15 years	Spouses felt like they were living with another person, given the marked changes in the personality of the stroke survivor Spouses felt that they had lost a life companion and mutual intellectual contact Spouses feared being confined at home due to the need to be always ready to help the stroke survivor Spouses struggled to find some time for themselves	Emotional challenges Role conflicts Marriage relationship: at a point of change
16	Satink, Cup, De Swart, & Sanden (2018) Nijmegen, The Netherlands	To explain how the partners of stroke patients described their own self‐management and their spouses’ management and how they have been supported postdischarge	Qualitative/focus group interviews were conducted	33 spouses of stroke patients participated Mean age of spousal caregivers: 59.2 years	At least 3 months poststroke	Stroke affected spousal caregivers; they found it challenging to care for their partner Spouses felt lonely, burdened, sad, and guilty Spouses used trial and error to self‐manage the poststroke situation and felt “lost” many a times Spousal felt that informal support would be useful	Emotional challenges
Study on stroke patients’ experiences and coping with spousal caregivers at home							
17	Thompson and Ryan (2009) Coleraine, Northern Ireland	To understand the impact of the consequences of stroke on spousal relationships from the perspective of stroke patients	Qualitative/semi‐structured interviews were conducted	16 stroke patients participated Mean age of stroke patients: 56 years	Mean length of time: 18 months poststroke	Spousal relationships were significantly affected in terms of sexuality, sexual desire, and sexual functioning poststroke. Stroke patients felt that they lacked control and that there was a dramatic change in how they perceived themselves poststroke	Emotional challenges Role conflicts

### 2.4 Ethics

This scoping review exercise was conducted as a desktop study. While statutory ethical approval was not required for such a study, it was carried out in an ethical manner.

## 3 RESULTS

Amongst the 17 studies that were included, nine were qualitative studies, one was a mixed‐methods study, and seven were quantitative studies. Amongst the seven quantitative studies, five were longitudinal studies, one was a cross‐sectional survey, and one was a cohort study. The included studies were conducted in six different regions or countries. More than half were conducted in Europe (*N = *10), namely the Netherlands (*N = *5), Sweden (*N = *2), Ireland (*N* = 1) and the UK (*N = *2), while the others were conducted in North America: the USA (*N = *4) and Canada (*N = *3). None of the studies included in the review were conducted in Asia.

Twelve out of the 17 studies focused on couples coping poststroke at home. The number of couples in the studies ranged from 2–211, with an average of 82 and a mean age of 60.8 years (range, 49.6–78.5 years old) (Nos. 1–12). Four studies focused on the experiences of spousal caregivers caring for stroke patients and their coping at home (Nos. 13–16). The mean sample size was 59 spouses (range, 7–180), with a mean age of 60.4 years old (range 53.3–74.3 years). The spousal caregivers had cared for their partner with stroke for 2 months to 15 years since the time of the stroke. One study focused on the stroke patients’ experiences, including those relating to coping with spousal caregivers at home (No. 17). That study included a total of 16 stroke patients with a mean age of 56, who had been living with stroke for an average of 18 months.

Five key themes were identified from the scoping review: (a) emotional challenges; (b) role conflicts; (c) lack of strategies in coping; (d) decreased life satisfaction of the couples; and (e) marriage relationship: at a point of change.

### 3.1 Theme 1 Emotional challenges

Thirteen reported on the emotional challenges faced by the stroke patients and their spousal caregivers (Nos. 2–4, 6–12, 15–17). These included feelings of emotional overwhelmment, stress, depression, loneliness, irritability and intolerance.

Five studies reported that the couples felt emotionally overwhelmed after the stroke event, which increased their sense of vulnerability (Nos. 2–4, 6, 9). “Vulnerability” was perceived by couples as a lack of control over the poststroke situation (No. 4). They lacked control due to uncertainty over the patient's prognosis; the demand for care because of the stroke patient's physical impairments; and a lack of information and support from healthcare professionals, which triggered feelings of distress, anxiety and frustration in the stroke couples (Nos. 2–4, 6, 9). Two studies examined the stress levels of stroke couples after the stroke patient had been discharged to go home from the rehabilitation hospital (Nos. 8, 10). In both studies, the stress levels of the couples were measured using the Perceived Stress Scale (PSS) and the Stress Appraisal Measure (SAM). Interestingly, stroke patients who were discharged to go home experienced lower levels of stress (mean score of 10.4, *SD* 7.30) than those who had been admitted to hospital (mean score of 12.3, *SD* 7.46) (No. 8). Spousal caregivers at home also experienced a slight decrease in their stress levels (mean score of 13.2, *SD* 7.12) than those caring for a partner in the hospital (mean score of 14.0, *SD* 7.25). Overall, spousal caregivers experienced higher levels of stress than the stroke patients, with a positive correlation between the PSS score of the stroke patient and that of the spousal caregiver (*p *< .01) (No. 8). Another study reported that the stress levels of spousal caregivers did not decline significantly over 6 months after the stroke patient was discharged home (No. 10). The results showed that when compared with stroke patients, spousal caregivers experienced higher levels of stress at home as a result of caring for their spouse with stroke over a period of 6 months to 1 year after the patient's discharge from a rehabilitation hospital.

Three other studies examined the manifestation of depressive symptoms in the spousal caregivers of stroke patients (Nos. 7, 11, 12). The symptoms were measured using the Patient Health Questionnaire‐9 (PHQ‐9) and the Goldberg Depression Scale (GDS). In one study, 51% of spousal caregivers were found to have experienced depressive symptoms (GDS ≥ 2) even 1 year after having been discharged to go home (No. 11).

Qualitatively, two studies reported on the spousal caregivers’ feelings of loneliness, helplessness, sadness, guilt and aggression as they struggled to comprehend their partner's poststroke behavioural changes and other symptoms (Nos. 15, 16). Both studies reported on the spouses’ sense of “loneliness and helplessness” and of being “lost” in that they felt as though they were caring for a “different” person after the stroke.

One study that explored the experiences of stroke patients reported that despite being conscious about their feelings of anger, irritability, agitation and intolerance towards their spousal caregivers, stroke patients were unable to control such emotional outbursts after the stroke (No. 17). This led to increased misunderstandings and arguments between couples, which further heightened the stroke patients’ guilt and despair. Overall, emotional challenges are common experiences of couples after a stroke.

### 3.2 Theme 2 Role conflicts

Five studies included in the review reported on the role conflicts between the stroke patient as the “care recipient” and the spouse as the “protector” after the stroke (Nos. 2, 4, 9, 15, 17). In these studies, male stroke patients felt conflicted about their masculine image and role as protector of the family, as they felt powerless about no longer being able to protect their family (No. 17). At times, they felt infantilized due to their spousal caregiver's hyper‐vigilance and overprotectiveness at home. This led to male stroke patients feeling sensitive about the change in their role to that of “care recipient” and about their inability to contribute financially to their family after a stroke (No. 4, 9). Male stroke patients grieved for their former self, who had been regarded by their wife as a “man” and “spouse” (No. 9).

### 3.3 Theme 3 Lack of strategies in coping

How stroke patients and their spousal caregivers coped after the stroke was reported in three studies (Nos. 10, 12, 14). These studies measured coping using the Revised Ways of Coping Questionnaire (R‐WCQ) (No. 10). Another study quantitatively reported on the coping of spousal caregivers, using the Utrecht Coping List (UCL) (No.12). The R‐WCQ measures five subscales of coping strategies, including coping through rationalization, hope, escape, openness towards others and giving control to others (No. 10). The UCL consists of seven subscales that measure the coping styles of active tackling, seeking social support, palliative reacting, avoiding, passive reacting, reassuring thoughts and the expression of emotions (No. 12).

Couples’ ways of coping with stroke at 6 months were determined using the R‐WCQ (No. 10). Stroke patients coped through “rationalization” and “giving control to others”, while spousal caregivers coped by “rationalization” and “openness towards others” (No. 10).

The coping styles of spousal caregivers were measured using the UCL (No. 12). It was found that spousal caregivers engaged in passive and avoidance coping at 3 years after a stroke. An example of avoidance coping included spousal caregivers “walking away” from caring for their partner with stroke (No. 12). Another study reported that spousal caregivers engaged in problem‐focused coping and the avoidance of emotional expressions when managing care related to practical tasks at home (No. 14). Spousal caregivers were found to suppress their emotions, disregard what they were feeling and focus on the tasks required to take care of stroke patients at home. Despite frustrations over their partner's inability to do some physical tasks at home, spouses continued to carry out the daily practical tasks of caring for the stroke patient at home (No. 14).

### 3.4 Theme 4 Decreased life satisfaction of the couples

Three studies explored the life satisfaction of couples living with stroke (Nos. 1, 11, 13). In all three studies, life satisfaction was measured using LiSat‐9 scores. One study reported that 48% of spousal caregivers were dissatisfied with life 1 year after the stroke of their partner (No. 11). Another study reported that more spouses (50%) were dissatisfied with life than stroke patients (28%), with a total LiSat‐9 score of ≤4 at 3 years after the stroke (No. 1). The third study reported that caregiver strain was positively associated with lower life satisfaction in spousal caregivers over 3 years after a stroke (No. 13). This is evidence that following a stroke the spousal caregiver has a lower level of life satisfaction than the stroke patient.

### 3.5 Theme 5 Marriage relationship: at a point of change

Three other studies reported that couples considered their marriage relationship to be at a point of change after a stroke (Nos. 2, 5, 15). One study reported that three couples who had been married for 11–15 years divorced after a stroke (No. 2). In this study, spousal caregivers felt that their efforts to care for their partner were not appreciated, while patients felt that their spouse lacked sensitivity towards their feelings and physical disability after the stroke. Stroke patients and spousal caregivers, who assumed that their partner was disengaging from the marriage relationship after the stroke, interpreted interactions with their partner in a negative light (No. 2). A quantitative study that measured positive relationship engagement between couples using the Mutuality Scale (MS) found that the mutuality scores of stroke partners decreased significantly at 1 year after a stroke (*F* = 15.56, *p *< .0001), which was attributed to a strained relationship (No. 5).

Another study reported that female spousal caregivers felt that “life was a struggle.” They were “tied” to the home while caring for their partner with stroke. They missed their female network and having time for themselves (No. 15). The female spousal caregivers negotiated with their partner to get some “time off for themselves.” The stroke partner was sent to a day rehabilitation centre or nursing home for a few days, with the promise of being picked up later. Overall, the stroke couples found their relationship to be at a point of change during the course of the stroke partner's recovery.

## 4 DISCUSSION

The most significant finding from this scoping review was that couples were unable to effectively cope with stroke in the community. Globally, an estimated 30 cases of stroke occur every 60 s; thus, a good understanding of stroke patients and their spousal caregiver's experiences and their ways of coping with stroke at home is essential. This would allow healthcare professionals to design appropriate supportive interventions to enhance the coping of couples in managing stroke survivorship at home (World Health Organization, 2018). The results of this review lead to the conclusion that after a stroke, stroke patients and their spousal caregivers face emotional challenges, role conflicts, ways of coping and decreased life satisfaction from managing at home. Although they had tried to cope with the stroke situation at home, the couples found themselves at a point of change in their married life. This information adds to the existing knowledge base about the coping of stroke patients and their spousal caregivers as couples, a subject that was often neglected in previous studies (Harris & Bettger, 2018; Lou, Carstensen, Jørgensen, & Nielsen, 2017). In addition, this scoping review sheds new light on the caregiving situation at home after hospitalization, from the perspective of the couples. Based on the current evidence, the time after the patient has been discharged to return home is a crucial period for addressing the education and support needs of stroke patients and their caregivers (Cameron & Gignac, 2008).

To the best of our knowledge, this is the first scoping review about how couples cope at home after a stroke. The emotional challenges that such couples face have included feelings of emotional overwhelmment, stress, depression, loneliness, irritability and intolerance (Anderson, Keating, & Wilson, 2017; Ekstam, Tham, & Borell, 2011; Green & King, 2009; Gosman‐Hedstrom & Dahlin‐Ivanoff, 2012; McCarthy & Bauer, 2015; McCarthy & Lyons, 2015; Ostwald, Bernal, Cron, & Godwin, 2009; Quinn, Murray, & Malone, 2014; Rochette, Bravo, Desrosiers, St‐Cyr Tribble, & Bourget, 2007; Satink, Cup, De Swart, & Sanden, 2018; Thompson & Ryan, 2009; Visser‐Meily, Post, van de Port, van Heugten, & van den Bos, 2008; Visser‐Meily et al., 2009). The results are quite similar to those of another review on couples coping with cancer (Traa, De Vries, Bodenmann, & Den Oudsten, 2015). Furthermore, we confirmed with other reviews that the emotional challenges experienced by stroke couples at home have been attributed to a lack of informational support and inadequate discharge preparation on stroke management by healthcare professionals (Forster et al., 2012; Peoples, Satink, & Steultjens, 2011). On the other hand, we found that being a couple and taking care of a spouse's well‐being may have a detrimental effect on the couple's relationship. Some recent studies on the well‐being of married couples have reported that chronic illness is a risk factor for divorce (Badr & Acitelli, 2017;Karraker & Latham, 2015).

This review identified different types of coping strategies that spousal caregivers adopted, such as “rationalization,” “being open towards others,” “passive coping” and “avoidance coping.” Existing studies have indicated that spousal caregivers who relied more on “active coping” and participated more in social activities experienced less stress than those who relied on other approaches (Forsberg‐Wärleby, Möller, & Blomstrand, 2004). It was further suggested that “active coping” maybe prove to be effective as a coping strategy for both stroke patients and their spousal caregivers in dealing with the poststroke situation (Visser‐Meily et al., 2009). If this is the case, more efforts should be made to encourage stroke couples to cope actively with their challenging situation.

It was found in other studies that the physical impairments of stroke patients impose changes in their role and that of their spousal caregiver. Male stroke patients experienced role conflicts when they can no longer acts a “protector” of their family at home (Taule & Råheim, 2014). This highlights the importance of education in addressing, prior to the patient's discharge, the potential challenges of conflicting ideas between stroke couples about the patient's role. It was suggested that female spousal caregivers should be made aware of creating opportunities for male stroke patients to contribute and participate in caring for themselves (Palmer & Palmer, 2011).

Other studies about spouses caring for patients with chronic diseases, such as Alzheimer's disease, reported that such spouses experienced caregiver burden and decreased life satisfaction over time (Joling et al., 2015). Similarly, we found in this review that while providing care at home spousal caregivers were more dissatisfied with life than the stroke patients. Therefore, interventions are needed to provide support to help spousal caregivers to cope and prepare to provide care at home (Rubbens, Clerck, & Swinnen, 2016). Li, Xu, Zhou and Loke (2015) systematically developed the Caring for Couples Coping with Cancer (4Cs) intervention programme to support couples in coping with cancer. The intervention was implemented in the hospitals before the patients were discharged to go home, with the aim of improving dyadic communication, dyadic coping, dyadic appraisal and quality of life. Besides the medical management of cancer, the 4Cs intervention emphasizes the importance of healthcare workers treating couples as “people” who require holistic support to cope after a family member has been diagnosed with cancer (Li, Xu, Zhou, & Loke, 2015). Another study examined the effects of a couple‐oriented intervention on patients with osteoarthritis (OA) and their spousal caregivers (Martire, Schulz, Keefe, Rudy, & Starz, 2007). The results of the study indicated that a couple‐oriented interventional approach comprised of support and education is valuable for spouses 6 months postintervention. Spousal caregivers experienced better outcomes in depressive symptoms and mastered their caregiver role after participating in the intervention, thereby indirectly benefiting the care delivered to patients with OA at home in the community (Martire et al., 2007). The various studies confirmed that couple‐oriented interventional approaches for patients and their spousal caregivers better prepare couples for their journey of coping with chronic health conditions.

Support interventions developed by medical and nursing healthcare professionals for couples with medical conditions have been found to have a positive impact on couples coping with home life after one of them has been discharged from the hospital. For example, a 12‐week group support intervention was designed for couples coping with a spouse's first diagnosis of myocardial infarction (Stewart, Davidson, Meade, Hirth, & Weld‐Viscount, 2001). The processes of the intervention included social comparisons, social learning and social exchanges, where emotional, informational and affirmational supports were rendered. The group support intervention had a positive effect on the couples’ coping, confidence, outlook in life and relationship with their partner (Stewart et al., 2001). Interestingly, another focus group study on the experiences of Chinese couples during convalescence from a first heart attack recognized the need for healthcare professionals to be better equipped to establish culturally sensitive support interventions for couples (Wang, Thompson, Chair, & Twinn, 2008). Compared with their Western counterparts, Chinese couples preferred to stay longer in bed and participated passively in exercise sessions that delayed their resumption of normal work on the patient's discharge from hospital. Throughout the phase of transitioning from hospital to home, Chinese couples reported feeling uncertainty and distress and had difficulties in adapting to changes in their lifestyle after a heart attack (Wang et al., 2008). Therefore, prior to developing an intervention, a further qualitative inquiry may need to be conducted to determine the personal values, cultural beliefs and practices in stroke management of stroke patients and their spousal caregivers. One study reported that the Indian Muslim community in South Africa believe that the occurrence of stroke is due to God's will (Bham & Ross, 2005). It is difficult to develop a culturally sensitive support intervention without much knowledge about how people of different ethnic groups experience and cope with the situation of stroke. Therefore, before any interventions are developed for stroke couples, a deeper understanding of the cultural beliefs of this target group should be taken into consideration to strengthen the development of a culturally sensitive intervention.

### 4.1 Limitations of the review

There are limitations to this scoping review, although it was intended to deepen our current knowledge related to the coping of stroke couples at home in the community. Only articles written in the English language were included. As such, the review might have missed important articles written in other languages. Although quantitative studies were included, some methodological weaknesses were evident. For instance, the generalizability of the quantitative results was limited in the longitudinal, cross‐sectional, cohort and mixed‐methods study designs. The studies included in the review also lacked a power analysis and an explanation of how the sample size was calculated.

### 4.2 Clinical relevance

The results of this scoping review contribute to knowledge pertaining to stroke couples coping at home in the community and have implications for both nursing policy and practice. It is critical to sufficiently prepare and support stroke patients and their spousal caregivers in their coping before the patient is discharged from hospital. The results of this review highlight the importance of carefully developing and implementing policies in hospitals to address the lack of support for stroke couples to effectively cope at home. Implementing support interventions for stroke couples in the nursing routine care plan would contribute to couples having more confidence in coping with stroke, even after the patient is discharged to go home from the hospital.

### 4.3 Implications for practice

- Nurse educators could play a pivotal role in guiding couples to cope with their stroke situation in hospital settings
- Ways of supporting stroke patients and their spousal caregivers could be included in the undergraduate, postgraduate and in‐service education to raise awareness amongst nurses. Further training would enable nurses to better support stroke patients and their spousal caregivers in their everyday professional life.

## 5 CONCLUSION

It is clear from this review that stroke is a life‐changing event for the patients and the spousal caregivers. Healthcare professionals have a significant role to play in supporting stroke patients and their spousal caregivers during the poststroke trajectory. This review discussed the importance of providing a supportive intervention to better prepare stroke patients and their spousal caregivers, prior to the patient's discharge from hospital. The intervention would be designed to help couples cope at home after a stroke. Such an intervention should be developed based on evidence and on the identified needs of the couples according to the Medical Research Council framework for developing complex interventions.

## CONFLICT OF INTEREST

The authors declare no conflicts of interest.

## AUTHOR CONTRIBUTIONS

SR, AYL and VCLC: Study design, data collection and data analysis. AYL and VCLC: Study supervision. SR: Manuscript writing. AYL and VCLC: Critical review for important intellectual content.
